# LSP-SPP Coupling Structure Based on Three-Dimensional Patterned Sapphire Substrate for Surface Enhanced Raman Scattering Sensing

**DOI:** 10.3390/nano13091518

**Published:** 2023-04-29

**Authors:** Shuqi Xie, Haipeng Si, Cong Liu, Weihao Liu, Muhammad Shafi, Shouzhen Jiang, Weiwei Yue

**Affiliations:** 1 Collaborative Innovation Center of Light Manipulations and Applications in Universities of Shandong, School of Physics and Electronics, Shandong Normal University, Jinan 250014, China; 2021020626@stu.sdnu.edu.cn (S.X.); lucky123018@163.com (C.L.); lwh990309@gmail.com (W.L.); shafiicp@gmail.com (M.S.); 2 Department of Orthopaedics, Qilu Hospital, Shandong University, Jinan 250012, China; 13065092736@163.com; 3 Shandong Key Laboratory of Medical Physics and Image Processing & Shandong Provincial Engineering and Technical Center of Light Manipulations, Jinan 250014, China

**Keywords:** SERS, 3D, photolithography, coupling, light-capturing, hydrophobicity, reproducibility, reproducibility

## Abstract

Although the fabrication of controllable three-dimensional (3D) microstructures on substrates has been proposed as an effective solution for SERS, there remains a gap in the detection and manufacturability of 3D substrates with high performance. In this study, photolithography is adopted to obtain a pyramid-like array on a patterned sapphire substrate (PSS), with Al_2_O_3_ as the dielectric layer. In addition, silver nanoparticles (AgNPs) are used to decorate Au films to obtain mass-producible 3D SRES substrates. In the case of low fluorescence, the substrate realizes the coupling of localized surface plasmon polaritons (LSPs) and surface plasmon polaritons (SPPs), which is consistent with the simulation results obtained using the finite element method. The performance of the SERS substrate is evaluated using rhodamine 6G (R6G) and toluidine blue (TB) as probe molecules with detection limits of 10^−11^ M and 10^−9^ M, respectively. The substrate exhibits high hydrophobicity and excellent light-capturing capability. Moreover, it shows self-cleaning ability and long-term stability in practical applications. Allowing for the consistency of the composite substrate in the preparation process and the high reproducibility of the test results, it is considered to be promising for mass production.

## 1. Introduction

Surface-enhanced Raman scattering (SERS) has developed rapidly in the past few decades due to its high sensitivity, which makes it widely applicable to structural analysis [1,2], adsorption interface surface state research [3,4], biomolecular interface orientation [5,6], etc. At present, there are two perspectives from which the mechanism of SERS enhancement is explored: the chemical enhancement mechanism and the physical enhancement mechanism. The former relies mainly on the chemical interactions between those atoms on the surface of the substrate and adsorbed molecules to manipulate the distribution of electron density, which can provide 10–100 enhancement factors [7]. The latter is attributable mainly to the enhancement of the local electric field on the metallic surface as a result of surface plasmon resonance (SPR), which is effective in significantly enhancing the Raman signal by up to 10^8^ times [8,9]. One of the most common ways to implement EM is the collective oscillation of free electrons of noble metals (Au, Ag, and Cu), which is usually performed in nanoparticle and tip gaps. This phenomenon is referred to as LSP [10,11]. Another solution to achieving this purpose is that free electrons are made to oscillate in the form of longitudinal waves along a metallic or dielectric layer, which is called SPP [12].

According to the above theory, the structure of nanoparticles and nano-films is essential for SERS. Since these structures are supported by the substrate, the microstructure of the substrate is equally important. 3D substrates have now attracted extensive attention for research because of their unique advantages in the number of hot spots, molecular trapping and optical properties [13]. To date, such 3D structures as nanoflowers [14], nanobowls [15], nanoantennas [16], nanopillars [17], and multilayered nanoparticles [18] have been widely explored. For periodic SERS substrates, however, low reproducibility and uncontrollable morphology make it difficult to mass produce these substrates. The three-dimensional substrates obtained by using chemical reduction methods are usually disadvantaged by aggregation effects. Moreover, due to the harsh conditions of the reaction, even slight environmental changes can have a significant impact on the quality of the substrate [19,20]. Zhang et al. applied a cost-effective yet efficient chemical method to construct a high-sensitivity polygonal silicon pyramid [21,22]. However, the uniformity of its metallic film was insufficient due to the annealing process. In addition, despite a significant improvement of uniformity for such pricey substrates as Klarite substrate with inverted pyramid array produced by Renishaw Diagnostics [23,24], their low sensitivity does not meet the demanding requirements in experiments due to their single mode of electromagnetic enhancement. Therefore, it is still necessary to produce a 3D substrate that can be mass-produced at low cost to achieve high sensitivity, excellent homogeneity and long-term stability. Active functional plasmonic technology has gained significant attention in recent years due to its potential for advanced sensing applications. For instance, Zhao et al. have demonstrated an innovative approach by designing a pyramidal nano-cavity structure that contains gold nanoparticles [25]. This unique structure is rich in electromagnetic hot spots, providing an excellent optical capture capability. In various studies, it has been suggested that photon scattering is increased when using patterned sapphire substrate (PSS), resulting in enhanced light extraction [26]. Kim et al. utilized wafer-level LSPR substrates by depositing Au films on patterned sapphire substrates and employed the prepared substrates for detecting biomolecules by observing LSPR shifts [27]. However, for 3D substrates such as PSS, a single metal film or particle cannot provide higher Raman enhancement. Nonetheless, the light-trapping ability of PSS is critical for SERS substrates and holds greater significance.

In some recent studies, it has been shown that the coupling effect of LSP and SPP can make difference in the dispersion relation and resonance spectrum [28]. Wind et al. calculated the coupling relationship between LSP and SPP by adopting the dipole model and mirror field theory [29]. Subsequently, the nanoparticle-metal film structure was widely applied due to its greater capability to excite stronger electric fields and generate wavelength shifts compared to single nanoparticle application [30,31]. However, in practice, there are high requirements for the horizontal spacing of nanoparticles and the spacing between nanoparticles and metallic films for SERS substrate. Due to the limitations of current technology, it is difficult to apply control on spacing in the plane, which restricts the improvement of sensitivity. However, it is feasible to achieve the vertical electric field enhancement of LSP and longitudinal wave coupling of SPP for those 3D substrates with controllable morphology [32]. In the meantime, the electromagnetic enhancement mode generated by this coupling places even more demanding requirements on the uniformity and stability of 3D substrates. We noted that in the process of LED preparation, some enterprises had produced PSS with a neat 3D shape such as the GaN substrate by combining photolithography and chemical etching. The patterned substrate as obtained by using the photolithography method shows a definite morphology and a consistent periodic array, which ensures the consistent quality of the substrate at the time of mass production. A new method to carry this out is to produce a nanoparticle–metal film and construct dielectric layer composite structures on PSS, which is conducive to improving the performance of higher quality 3D substrates through stronger electromagnetic enhancement. This ensures the reproducibility and long-term stability of industrial production. In addition, 3D structure is usually accompanied by the improvement of hydrophobicity, which improves the detection of solution molecules.

In the present study, a facile method to mass produce 3D Ag NPs/Au/Al_2_O_3_ composite substrates is proposed. The periodic pyramid-like arrays on the PSS as obtained by the lithographic method enable the three-dimensional distribution of Ag NPs in multiple directions and across a larger surface area. In addition, a richer “hot spot” is provided by the cavity gaps caused by the 3D structure. Al_2_O_3_ is treated as the dielectric layer due to its capability to remove the charge disorder of the medium while reducing the spectral diffusion of excitons. Using the finite element method, the excitation and coupling of LSP and SPP are demonstrated by simulating the electric field distribution of the substrate. The composite structure substrate exhibits high hydrophobicity and light-harvesting ability, with the limits of detection reaching 10^−11^ M and 10^−9^ M for R6G and TB, respectively. Apart from that, it is capable of self-cleaning and recycling. The high uniformity and high reproducibility evidenced by the experimental results illustrate the applicability of this composite substrate in industrial mass production.

## 2. Materials and Methods

### 2.1. Materials

Ethanol (C_2_H_6_O, 99.7%) and acetone (CH_3_COCH_3_, 99.5%) were purchased from Tianjin Fuyu Fine Chemical Co., Ltd. (Tianjin, China). Rhodamine 6G (R6G, MW = 479.02) was sourced from Shanghai Zhanyun Chemical Co., Ltd. (Shanghai, China) Moreover, toluidine blue (TB, MW = 373.97) was obtained from Sinopharm Chemical Reagent Co., Ltd. (Shanghai, China).

### 2.2. Preparation of SPP Composite Structures Composed of Au/Al_2_O_3_/Ag NPs

**Figure 1:**
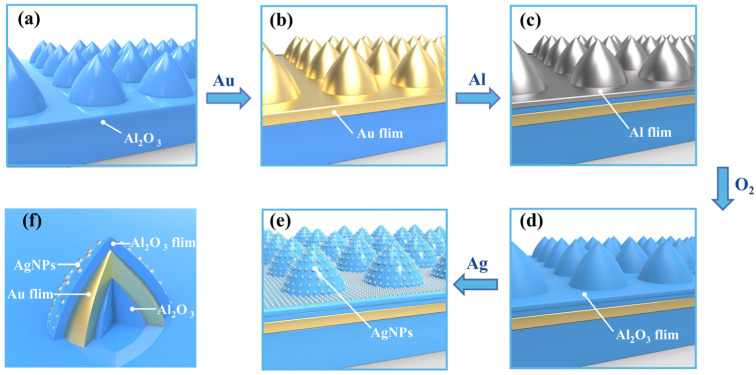
(**a**–**e**) Schematic flow diagram of PSS composite structure. (**f**) Cross section of the pyramid-like microstructure.

The surface of the PSS was initially cleaned using ultrasonic waves. Then, acetone and ethanol were used in turn to clean the substrate in a cleaning machine for 20 min, and rinsed with deionized water (DI water) to protect the surface from contamination. At a low pressure of 7 × 10^5^ Pa and a current of 55 A, a uniform and continuous layer of 50 nm thick Au was deposited on PSS at a rate of 0.1 Å/s. Next, 3 nm of Al was deposited on the surface of the Au film given a current of 75 A, the same gas pressure and a rate of 0.8 Å/s. Oxygen was continuously infused into the closed vacuum reactor, and the air pressure was adjusted to 1 × 10^3^ Pa. The Au/Al PSS structure was placed in the reactor for 10 min to fully oxidize the surface Al film and form the first layer of Al_2_O_3_ film through a reaction with oxygen. Subsequently, 3 nm of Al was deposited again under the same conditions, and the oxidation process was repeated to form a second layer of Al_2_O_3_ film. Finally, 3 nm of Ag was deposited on the surface of Al_2_O_3_ at the pressure of 7 × 10^5^ Pa and a current of 60 A. Under these conditions, dense silver hemispherical nanoparticles formed on the surface of the multilayer film. The experimental process is shown in Figure 1.

### 2.3. Characterization of Substrate Morphology and SERS Detection

The three-dimensional structure, multilayer film structure and nanoparticles on the substrate surface were observed under SEM (Zeiss GeminiUltra-55, Baden-Württemberg, Germany). The chemical composition of the samples was determined using EDS. Taken as the probe molecule, R6G was dissolved in ethanol to obtain the solutions of different concentrations (10^−6^–10^−11^ M). Then, with the substrate split into several samples for testing, 2 µL of R6G solution was taken for titration and analyzed by Raman spectrometer (Horiba HR Evolution, Kyoto, Japan) after drying. As for TB aqueous solution, the same method (532 nm wavelength laser, single sampling time 2 s, 0.048 mW power) was adopted for SERS signal detection.

## 3. Results and Discussion

### 3.1. Structure Characterization

**Figure 2:**
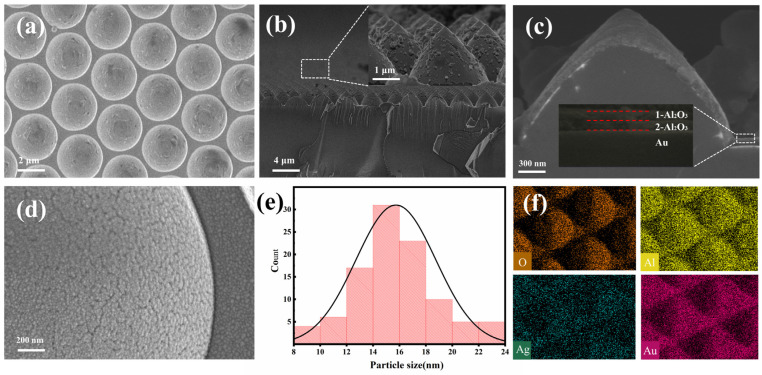
(**a**) SEM top view and (**b**) front view. The inset is an enlarged view of the part. (**c**) The cross-sectional SEM image of the composite SERS substrate. (**d**,**e**) SEM images and size distribution histograms of Ag NPs. The inset is an enlarged view of the part. (**f**) EDS element diagram of different elements.

In order to verify whether the PSS composite substrate was of satisfactory quality, its morphology was characterized by SEM. Figure 2a,b show the surface and cross-section of the substrate, respectively. The substrate consists of pyramid-like units arranged in an orderly manner, each having the same parameters. Figure 2c shows the profile of the smallest element on the composite substrate. It can be seen from the figure that Au and Al_2_O_3_ form a dense and continuous film on the surface. The interface between Au film, Al_2_O_3_ film and the Al_2_O_3_ prepared twice can be observed by magnifying the fault. Figure 2d,e show the distribution of silver hemispherical nanoparticles on the substrate surface and the histogram of their size distribution. Among these 100 samples of nanoparticles, the range of 12–18 nm is the main interval of diameter distribution, which basically shows a typical Gaussian distribution. Due to the silver hemispherical nanoparticles obtained by using the thermal evaporation method, the integrity of the microstructure and its overall uniformity are ensured, which allows the ideal hot spots to be excited. EDS is used to map the surface of the composite substrate. The composition of various elements (O, Al, Ag, and Au) is depicted in Figure 2f. The continuous distribution of elements reaffirms the uniformity of silver nanoparticles and films, indicating the successful construction of the composite structure.

### 3.2. Simulation and Theory

**Figure 3:**
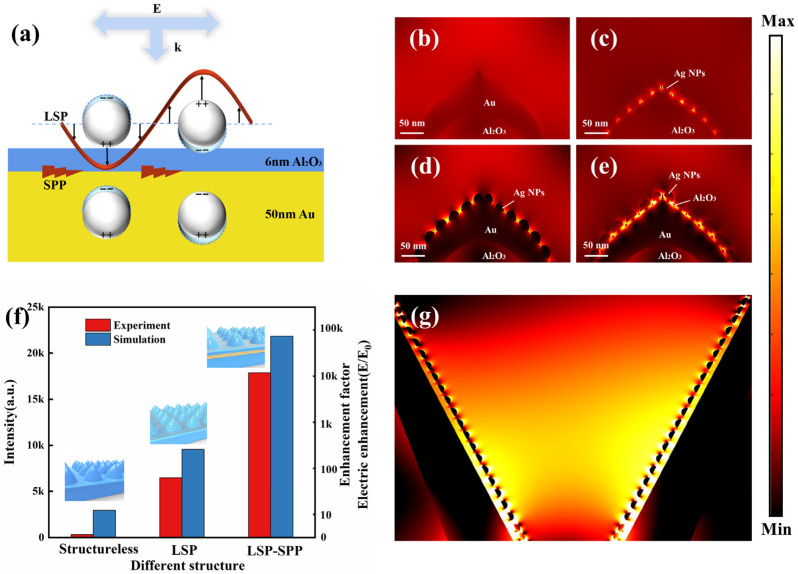
(**a**) LSP-SPP coupling model. (**b**–**e**) Electric field distribution for different structures at 532 nm wavelength of exciting light: (**b**) PSS and Au/Al_2_O_3_, (**c**) PSS and Ag NPs, (**d**) PSS and Ag NPs/Au, (**e**) PSS and Ag NPs/Au/Al_2_O_3_. (**f**) The variation in electric field enhancement (E/E_0_) and the Raman intensity of R6G at 613 cm^−1^ for the different structures. (**g**) Bottom cavity array electric field.

In order to further reveal the coupling mechanism of LSP and SPP, the models of Ag NPs, Au film and interval layer (Al_2_O_3_) are constructed, as shown in Figure 3a. The LSP in the composite system results from the superposition of LSP as produced by individual metallic nanoparticles, which SPP propagates along the metallic surface. When LSP matches SPP in the conditions of excitation, Ag NPs induce a mirror dipole with an opposite charge in the metallic film. The redistribution of induced charges makes the potential difference more significant. The electromagnetic field is severely constrained in the gap. Due to the near-field coupling effect, the surface of metallic film frees charges to form a significantly enhanced electric field. The existence of the interval layer is effective not only in restricting the coupling distance between LSP and SPP, but also in preventing the loss of electromagnetic field in the air gap.

**Figure 4:**
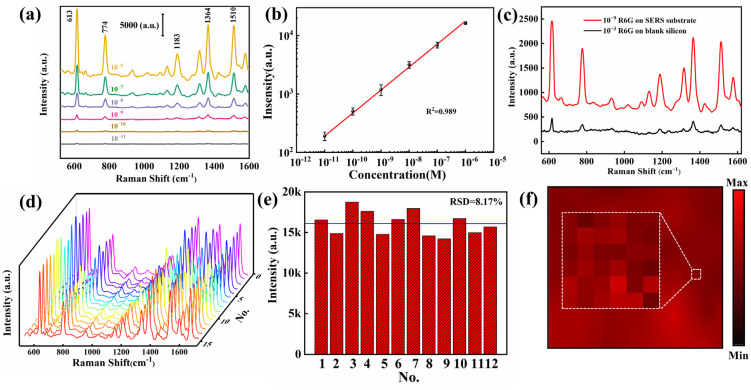
(**a**) Raman spectra of R6G on the composite SERS substrate with different concentrations (10^−6^ M to 10^−11^ M). (**b**) SERS intensity at 613 cm^−1^ for R6G as a function of the molecular concentration. (**c**) Raman spectrum of 10^−9^ M R6G on SERS substrate and that of 10^−3^ M R6G on Si substrate. (**d**) 15 Raman spectra of R6G at a concentration of 10^−6^ M are randomly collected from the substrate. (**e**) The average Raman intensity of R6G at 613 cm^−1^ from 12 different batches of the SERS substrates. (**f**) Local uniformity test results of 100 sampling points within the rectangle.

In order to better understand the electric field distribution of the PSS composite structure and to establish whether the LSP and SPP are successfully coupled, the finite element method is used to perform simulation. The parameters were based on the SEM characterization results. The electric field distribution is simulated for 4 different combinations, respectively. By comparing the electric fields shown in Figure 3b,c, it can be found that the SPP fails to be excited when there are only metallic and interval layers on the PSS, as indicated by the weak electric field. This is because the wave vector of SPP is higher than the wave vector of light at all times given the same frequency [33]. As a result, it cannot be excited directly by the incoming light in the air, whereas Ag NPs lead to the uneven distribution of charges under the disturbance of external electromagnetic waves. That is to say, under the electromagnetic excitation of the incident light, the electronic oscillation frequency is identical to that of the excitation light, which meets the condition of momentum matching of LSP. Moreover, the structure exhibits strong hot spots. When Ag NPs are combined with Au film, the system becomes asymmetric, with the corresponding mirror image dipoles generated in the Au film. Due to the coexistence of Ag NPs and dipoles, there are a large number of opposite charges accumulating between the gold film and the Ag NPs. This gives rise to a potential difference. Then, a strong electromagnetic field is generated in the gap. As an interval layer, Al_2_O_3_ controls the coupling distance and divides the Ag NPs and Au films into cavity-like structures [34]. As a result, there is a stronger “hot spot” in Figure 3e than in Figure 3d. As shown in Figure 4f, the Raman test results of different structures are compared with the enhancement factors obtained by electric field simulation. It can be found that the simulation results are highly consistent with the experimental results, suggesting that the substrate performance can be significantly improved by the coupling of LSP and SPP. In addition, the micrometer-tapered cavities and micrometer trumpet-shaped cavities demonstrate outstanding electrical and optical properties. When SPP is conducted along the metal surface in the form of waves, the electromagnetic wave conducted from adjacent structures to the bottom interferes with each other due to the difference in phases, thus resulting in the enhancement of the electric field. The periodic arrays create a series of cavities at the base of the pyramid-like structures, enhancing the substrate’s light-harvesting capabilities and leading to an expansion of “hot spots” [35,36]. This is manifested as the reduced light reflectivity of the substrate and the focusing electric field caused by the concentration of incident light at the bottom, according to Figure 3g.

### 3.3. Performance Exploration

To further explore the surface-enhancing ability of the substrates, the R6Gs of different concentrations (10^−11^–10^−6^ M) were titrated on the substrates and Raman tests were performed. As shown in Figure 4a, the intensity of the SERS signal is proportional to the concentration of R6G, with distinct signal peaks observed at 613, 774, 1183, 1364, 1510 and 1651, respectively. When the concentration of R6G declines to 10^−11^ M, the signal is low in intensity and the peaks are observable only at 613 and 774, indicating that the limit of substrate detection is reached. In order to ensure the reliability of the data, 10 sampling points were taken from the samples with different R6G concentrations. Additionally, a linear relationship between the signal intensity and concentration was fitted, as illustrated in Figure 4b.

A quantitative analysis of substrates is conducted using computational enhancement factors:

(1) EF=ISERSNSERSIRamanNRaman

where *I_SERS_* denotes the intensity of the peak of 10^−9^ M R6G at 613 cm^−1^ on the composite SERS substrate; *I_Raman_* indicates the intensity of 10^−3^ M R6G at the same wavelength on the silicon substrates as shown in Figure 4c; *N_SERS_* and *N_Raman_* represent the number of probe molecules accumulating in the laser spot on the composite SERS substrate and the silicon substrates, respectively. 2 μL of R6G is titrated on PSS substrate and silicon substrate, respectively. Then, the formed dispersion area and laser spot area are measured and calculated. The final EF [37] is calculated to be 7.2 × 10^7^, as detailed in the supplement to this paper. In addition to quantitative analysis, the widely used SERS substrates are also required to have excellent uniformity and reproducibility. To ensure the accuracy of results, 15 sampling points are randomly selected on the R6G substrate of a 10^−6^ M concentration. The Raman signal corresponding to each concentration is taken as the mean value of 15 sampling points. According to Figure 4d, the data is highly consistent. Furthermore, the reproducibility of the substrate during preparation is evaluated by collecting the characteristic peaks from 12 batches of samples, as depicted in Figure 4e. The adjacent sampling points are selected on the substrate, and the color of the rectangular range represents the results of the Raman test. To assess the uniformity of the substrate, 100 sampling points are selected on the substrate, with the color depth in the rectangular range indicating the size of the peak value. As can be seen from Figure 4f, the substrate exhibits high local uniformity. The results mentioned above indicate that the mass-produced PSS composite substrate can preserve the high uniformity of the SERS signal, thereby guaranteeing high reproducibility despite minor manufacturing errors.

**Figure 5:**
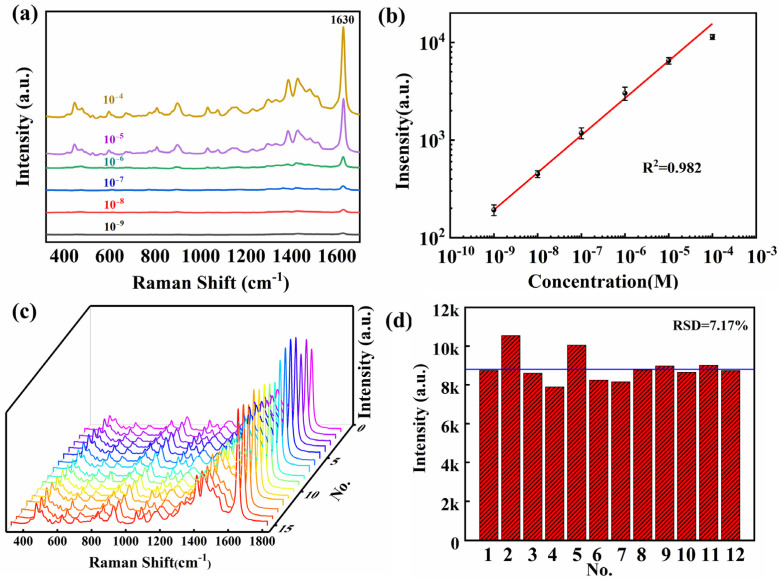
(**a**) Raman spectra of TB on the composite SERS substrate with different concentrations (10^−9^ to 10^−4^ M). (**b**) SERS intensity at 1630 cm^−1^ for TB as a function of molecular concentration. (**c**) 15 Raman spectra of TB at a concentration of 10^−4^ M are randomly collected from the substrate. (**d**) The average Raman intensity of TB at 1630 cm^−1^ from 12 different batches of the SERS substrates.

As a quinoneimine dye containing multiple chromophores and auxochromes, TB [38,39] is commonly applied as a biochemical probe in cells. Clinically, it has been adopted to diagnose such symptoms as oral cancer and diphtheria, playing an important role in the nucleic acid test. To evaluate the clinical viability of the SERS substrate, TB is employed to evaluate the substrate’s efficacy in detecting toxic substances. 2 μL of TB aqueous solutions of different concentrations (10^−9^–10^−4^) are titrated on the substrate, and the SERS spectra are shown in Figure 5a. There is a reduction in the intensity of the characteristic Raman peak of TB (1630 cm^−1^) when the concentration of solution decreases given the detection limit of about 10^−9^ M. As shown in Figure 5b, there is a typical linear relationship between the intensities of Raman peaks at different concentrations. According to Figure 5c, a comparison is drawn in the characteristic peak intensities of the above 15 TB spectra. The blue dot represents the relative intensity of the peak at 1630 cm^−1^, whereas the red dashed line indicates the average intensity with a relative standard deviation (RSD) of 7.17%, as shown in Figure 5d. These results demonstrate that this substrate applies to the detection of probe molecules, as well as biochemical and medical detection.

### 3.4. Hydrophobicity Characterization and Self-Cleaning Test

**Figure 6:**
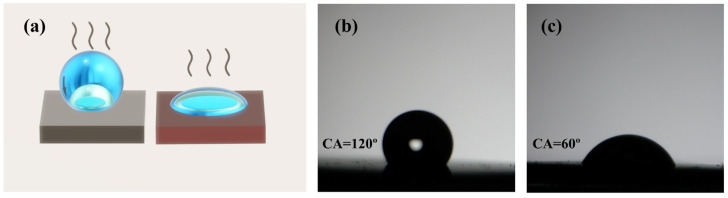
(**a**) Enrichment of hydrophobic substrates for molecular detection in the solutions. (**b**) PSS hydrophobicity characterization. (**c**) Planar Al_2_O_3_ hydrophobicity characterization.

As suggested in studies, substrates with high hydrophobicity can be used to enrich solutes when solutions are detected, which is beneficial to the detection of trace molecules in liquids. According to Figure 6a, the liquid of the same volume to be tested on different hydrophobic substrates shows different evaporation modes under the limitation of tension, and there is a significant difference in the remaining area of the test object on the substrate. By collecting more test objects in a more confined area, the acquisition of optical and electrical signals can be facilitated, which is conducive to Raman detection [40,41]. Figure 6b,c illustrate the hydrophobicity characterization of the substrate. Compared with the planar Al_2_O_3_ of the same composite structure, the PSS composite structure shows significant hydrophobicity. Based on the characterization results, the 3D microstructure proves to be effective in imparting hydrophobicity to the initially hydrophilic surface. In addition, high hydrophobicity is essential for the detection of oil-water mixtures and the long-term storage of substrates, which makes it suitable for practical applications.

**Figure 7:**
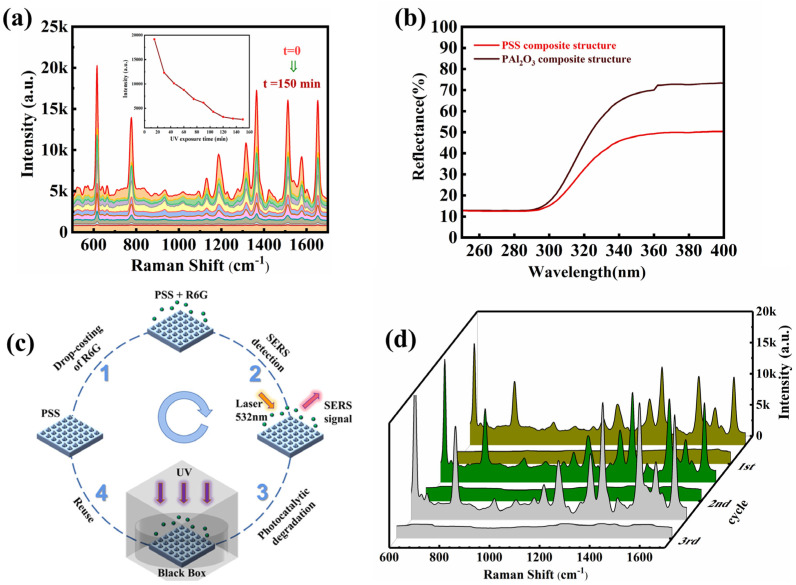
(**a**) R6G Raman spectra of PSS composite substrates irradiated at UV intervals for 150 min. (**b**) UV reflectance of PSS composite substrates and planar-Al_2_O_3_ composite substrates. (**c**) Recycling experimental process of SERS substrate. (**d**) The SERS spectra of R6G after the illumination of UV for 3 cycles.

The self-cleaning capability of the substrate not only reduces the production cost but also prevents the destruction of the substrate’s surface structure during manual cleaning processes. Figure 7a shows the decreasing trend displayed by the signal intensity of the characteristic peak for the 10^−6^ M solution of R6G under intense UV irradiation over time. It can be seen from the figure that R6G continues degradation over a period of 150 min and reaches the detection limit of Raman intensity after one period. According to the Langmuir-Hinshelwood theory, the UV photons and photo-induced carriers on PSS can be used to control the surface degradation reaction. Take the photolysis process of R6G as an example. The self-cleaning process of the substrate is evidenced in two aspects. On the one hand, it has been demonstrated previously that Ag NPs can catalyze the degradation of small organic molecules [42]. On the other hand, the microstructure of PSS can significantly improve the light-capturing ability. As shown in Figure 7b, a comparison is performed in the reflectivity of the plane Al_2_O_3_ composite structure and the PSS composite structure to the light in the ultraviolet band. The latter improves the utilization of light, thus promoting the self-cleaning process of R6G molecules. To further evaluate the self-cleaning capability of the substrate, three cycles of R6G molecular detection and three self-cleaning cycles are performed on the same substrate, as shown in Figure 7c. The reused substrate exhibits a comparable detection capability to the brand-new substrates, and there is a significant reduction in the intensity of background fluorescence, as shown in Figure 7d. The above results can be applied to measuring a broader range of molecules, thus providing a valuable reference for substrate recycling.

### 3.5. Outlook and Development

The 3D PSS composite substrate proposed in this article has great potential for application in fields such as biological detection, environmental monitoring, chemical analysis, etc. Therefore, how to increase the applicability of the substrate is the focus of future work. Uddin et al. used a metal–dielectric–metal structure instead of a single metal layer on a typical SPCE glass prism used in fluorescence microscopy, which can significantly improve the sensitivity of SPCE based fluorescence microscopy systems, which will help enhance single molecule detection [43]. Rai et al. used solidus as a reducing and capping agent to obtain Ag NPs, Au NPs, and Ag-Au nanocomposites, and constructed a plasma coupling model for spacer, activity, and extended activity nanoconfigurations, which played an important role in the development of SPCE bioplatform sensors [44]. Similarly, building molecular monitoring platforms on SERS substrates with excellent properties will have great development prospects. Additionally, in the experiment, it is possible to construct a core–shell structure using ZIF-8 and Au NPs to form a MOF hybrid. Using these MOF structures to further improve the SERS platform can achieve system specific recognition and improve monitoring sensitivity.

## 4. Conclusions

To conclude, the current study proposes a novel method for constructing a 3D composite structure on PSS, and the electric field distribution of the structure is theoretically simulated to demonstrate its enhanced LSP-SPP coupling mechanism, which effectively enhances the electric field. Moreover, the EF of the composite SERS substrate is close to 7.2 × 10^7^. According to SEM and EDS results, the substrate shows controllable and uniform morphology in the production process. When the concentration of the R6G solution reaches as low as 10^−11^ M, the SERS signal remains detectable, and the detection limit is 10^−9^ M for the TB. The substrate displays excellent hydrophobicity, with the liquid contact angle reaching 120°. In the self-cleaning test, the substrate can be reused after 150 min of UV irradiation, maintaining low background fluorescence after three cycles. Due to the high reproducibility and uniformity of the substrate as revealed in the above experiments, it can be mass-produced in practice.

## Data Availability

The data are contained within the article.
